# Psychological consequences of female genital mutilation: A mixed-method systematic review

**DOI:** 10.4102/sajp.v79i1.1877

**Published:** 2023-07-07

**Authors:** Tara Reman, Valerie Balligand, Benoit Schoefs, Veronique Feipel, Jeanne Bertuit

**Affiliations:** 1 School of Health Sciences (HESAV), Lausanne, Switzerland; 2 Laboratory of Functional Anatomy, Faculty of Medicine, Université Libre de Bruxelles, Brussel, Belgium; 3 Department of Health, Haute Ecole Libre de Bruxelles Ilya Prigogine, Bruxelles, Belgium; 4 CHU St-Pierre, Clinique du Périnée, Brussel, Belgium

**Keywords:** female genital mutilation, cutting, mental health, psychological symptoms, mixed method systematic review

## Abstract

**Background:**

Female genital mutilation (FGM/C) defined as ‘all procedures that involve partial or total removal of the external female genitalia, or other injury to the female genital organs for non-medical reasons’ is a cultural practice having several consequences on women’s health. Medical and sexual consequences have been documented, but the link between FGM/C and the development of psychological symptoms is not clearly established. The influence of contextual factors is poorly understood.

**Objectives:**

To evaluate the psychological impact of FGM/C and how victims experience it.

**Method:**

A mixed method systematic review was conducted. The inclusion criteria were observational primary studies involving women who had undergone FGM/C and had experienced psychological symptoms. Publication bias was assessed by using the Mixed Methods Appraisal Tool. A configurative strategy that involved a comparison of quantitative and qualitative data was used, followed by an analysis of causal link between FGM/C and induced psychological disorders.

**Results:**

Fourteen studies were included. Post-traumatic stress disorder (PTSD), depression, anxiety and somatisation showed a significantly higher prevalence in women who have experienced FGM/C versus non-mutilated women. Female genital mutilation type II or III were identified as predictors of disorder severity. Qualitative studies showed a significant difference in the perception of FGM/C between immigrant and non-immigrant women, as well as the multidimensional nature of the factors influencing disorders’ onsets.

**Conclusion:**

Our study showed a high association of FGM/C (and its degree of severity) with psychological disorders such as PTSD, depression, anxiety and somatisation. It also illustrates contextual factors, including socio-cultural factors that may influence the intensity of these psychological disorders.

**Clinical implications:**

It is important for health professionals to be aware of the psychological consequences of FGM/C and the different factors influencing FGM/C perception. Indeed, a feeling of ‘Being abnormal’ can be awakened among patients because of health professionals’ incorrect behaviours.

## Introduction

Female genital mutilation or circumcision (FGM/C) is defined as ‘all procedures that involve partial or total removal of the external female genitalia, or other injury to the female genital organs for non-medical reasons’ (Haut Commissariat des Nations Unis des Droits de l’Homme et al. 2008). Worldwide, 200 million women have undergone FGM/C and the number of potential victims is estimated at 3 million each year (World Health Organization 2020). There are four type of FGM/C depending on the level of damage to the female external genitalia (HCDH et al. 2008): (1) removal of the clitoris (partial or total); (2) removal of the clitoris and labia minora (partial or total); (3) narrowing the vaginal opening (infibulation) or (4) any non-medical harmful practice for example, burning or pricking. The customs of the communities where FGM/C is practised encourage the perpetuation of these acts of violence against women for cultural or symbolic reasons (Andro & Lesclingand 2016). Some of these customs include beliefs that FGM/C can increase childbirth ability, ensure chastity, prevent promiscuity of women and girls and/or meet religious requirements (Berg, Denison & Fretheim 2010; Terry & Harris 2013). Additionally, women who were not mutilated face ostracisation and socio-economic hardship (Berg et al. 2010) as well as stigmatisation (Terry & Harris 2013). In addition to the acute and chronic genitourinary complications, FGM/C has a significant psychological impact on its survivors. Previous quantitative findings have indicated a correlation between FGM/C and mental disorders such as post-traumatic stress disorder (PTSD), depression, anxiety and sleep disorders (Behrendt & Moritz 2005; Chalmers & Hashi 2000; HCDH et al. 2008; Mulongo, Mcandrew & Martin 2014). Other mental disorders like somatisation and phobia are mentioned (Elnashar & Abdelhady 2007). However, some authors criticise the simplistic quantitative analysis made, disregarding the impact of contextual factors such as the type of FGM/C or the migrant status of mutilated women (Pastor-Bravo, Almansa-Martínez & Jiménez-Ruiz 2018). The inclusion of qualitative data would provide a broader overview of the factors influencing the development of these psychological disorders and would lead to clear recommendations to establishing multidisciplinary treatment guidelines.

To bridge this gap, we conducted a mixed-method systematic review to map out existing literature on FGM/C’s psychological impact, based on both quantitative and qualitative data. The review sought to address the following research questions: (1) What are the main psychological disorders induced by FGM/C? and (2) How are these disorders explained by women who have undergone FGM/C?

## Methods

Our mixed method systematic review followed the ‘Convergent Segregated’ methodological framework proposed by the Joanna Briggs Institute (JBI) and the Preferred Reporting Items for Systematic Reviews and Meta-Analyses (PRISMA) statement.

### Information sources and search strategy

We searched the following electronic databases from September 2020 to December 2020: PubMed, CINAHL, PsychINFO and EMBASE. Guided by PICO, a search equation was developed by selecting Medical Subject Healing (MeSH) keywords and was adapted to the thesaurus of each database. The Boolean logic was adopted, such as ‘circumcision, female’ or ‘FGM’ or ‘female genital cutting’ or ‘female genital mutilation’ or ‘female excision’ or ‘clitoridectomy’ or ‘infibulation’ and ‘stress disorders, post-traumatic’ or ‘depression’ or ‘anxiety’ or ‘somatoform disorders’ or ‘adjustment disorders’ or ‘affective disorders, psychotic’ or ‘adaptation, psychological’ or ‘body dissatisfaction’ or ‘PTSD’ or ‘post-traumatic stress disorder’ or ‘insomnia’ OR ‘chronic pain’ or ‘depression’ or ‘anxiety’ or ‘coping’ or ‘psychological effects’ or ‘psychological consequence’ or ‘mental health’ or ‘psychosocial consequence’.

### Inclusion criteria

Qualitative, quantitative and mixed-methods primary observational studies relating to psychological consequences of FGM/C for women were considered. Only studies published between 2010 and 2020, in French and English were included.

Studies were included if they involved women over 13 years old who had previously undergone FGM/C (type I, II and III) and experienced psychological disorders like *PTSD* defined as an anxiety disorder developed after a traumatic event, depression as persistent sadness and a lack of interest or pleasure in previously rewarding or enjoyable activities, *anxiety* characterised by feelings of tension, worried thoughts and physical changes, *somatisation* which involves one or more physical symptoms accompanied by an excessive investment of time, energy, emotion and behaviour related to the symptom that results in significant distress and *sleep disorder*, were considered. Mental disorders had to be evaluated with validated questionnaires like General Health Questionnaire (GHQ-28), PTSD Check List-Civilian Version (PCL-C) and Hopkins Symptom Checklist-25 (HSCL-25). There were no limitations on country or social group.

### Study screening

The selected studies were imported into Mendeley^®^ and the duplicates were removed. The remaining studies were screened for relevance based on title, abstract and then underwent full-text screening against inclusion criteria. Screening was conducted by two authors (V.B. and J.B.).

### Critical appraisal

The Mixed Methods Appraisal Tool (MMAT) was used to critically appraise the literature. It is a validated and reliable tool that offers criteria specific to observational and interventional study design, including quantitative and qualitative designs (Hong et al. 2018; Pace et al. 2012). No study was excluded based on MMAT scores. Evaluation was conducted by one author (T.R.).

### Data collection

Data extracted from included studies comprised demographic characteristics (sample size, age, age at time of FGM/C, FGM/C’s type, countries of origin and immigration status, residential area, education level, other trauma), study design, outcomes and measurement tool for quantitative studies. For qualitative studies and mixed studies: demographic characteristics (same as quantitative studies); study design; data collection; data analyses and outcomes were extracted.

### Data analysis and synthesis

Quantitative and qualitative studies’ results were integrated following a ‘configurative analysis’ involving a systematic comparison of quantitative and qualitative data followed by an analysis of causal link between FGM/C and induced psychological disorders. Initial analysis was conducted by V.B. and validated by J.B.

## Results

### Study selection

**FIGURE 1:**
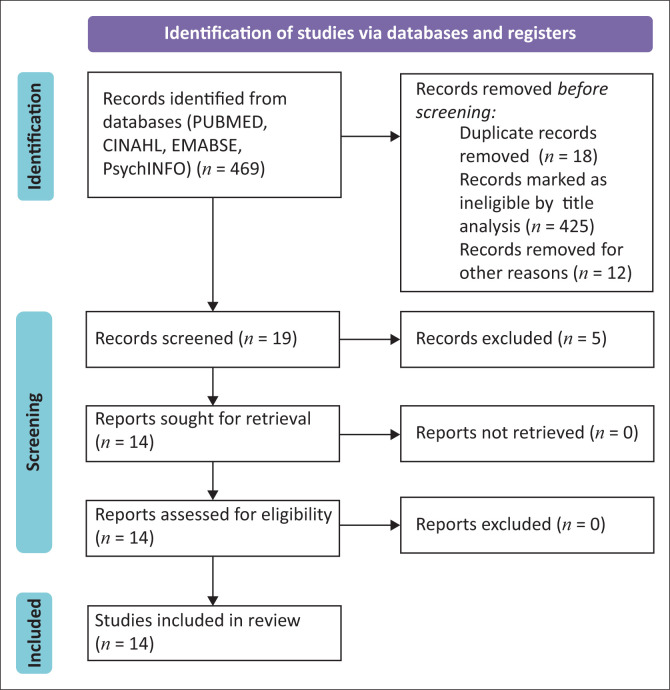
Preferred Reporting Items for Systematic Reviews and Meta-Analyses flowchart.

We identified 469 records from electronic databases (Pubmed: *n* = 145, CINAHL: *n* = 47, PsychINFO: *n* = 252 and EMBASE: *n* = 25). Out of these records, 432 were not eligible based on the title and/or abstract, 5 were not eligible based on the full text and 18 were duplicates. A total of 8 quantitative, 4 qualitative and 2 mixed method studies were included. All were published between 2010 and 2020. Figure 1 illustrates the PRISMA flow chart with the reasons for exclusion.

### Characteristics of the studies and data extracted

Six out of the 14 observational studies were designed as cross-sectional studies, other designs included case control (*n* = 2), cohort (*n* = 1), retrospective (*n* = 1) and qualitative (*n* = 4) studies. Study characteristics and data extracted (population, design, research method, outcomes, sample size) are summarised in Table 1 and Table 2.

**TABLE 1:** Summary of quantitative and mixed method studies’ characteristics.

Authors and date	Design	Sample size	Measurement tool	Outcomes
**Quantitative study**				
Piroozi et al. 2020	Observational case control study	***n* = 247** *M*: 122 (49%) nn-*M*: 125 (51%)	General Health Questionnaire (GHQ-28)	Anxiety, somatisation insomnia, social dysfunction, depression
Im et al. 2019	Observational cross-sectional study	***n* = 143** *M*: 57 (39.8%) nn-*M*: 86 (60.2%)	PTSD Check List – Civilian Version (PCL-C);Hopkins Symptom Checklist-25 (HSCL-25) and 7 added items for somatisation Psycho-social disorders: Revised Attitudes Towards Violence Scale; Adapted Social Capital Assessment Tool; added items for other psycho-social factors	Presence of poly-victimisation (exposure to multiple traumas: pre- and post-immigration, family trauma, individual trauma) PTSD, depression, anxiety, somatisation, drug use, suicidal thoughts, physical health, psychosocial factors (socialisation problems, acceptance of violence, sense of community, help-seeking, emotional coping, problem-solving, mental health awareness and psychosocial needs)
Kobäch et al. 2018	Observational cross-sectional study	***n* = 165** *M*: 147 (89.0%) nn-*M*: 18 (11.0%)	PSS-I, assessing 17 PTSD symptom criteria (range 0–51) ShuD Shutdown Dissociation Scale (13 items) Hopkins Symptom Checklist-25 (HSCL-25) Mini International Neuropsychiatric interview (M.I.N.I.; subscales A, B, K and L.) Hair Cortisol Concentrations (HCC) – measure neuroendocrinologqiue	PTSD diagnosis and ShuD score total score adding sub-scores of transient deafness or blindness, nociception, analgesia, numbness, transient paralysis, loss of language, pseudo-neurological syndrome, (pre)syncope and out-of-body experience HSCL and M.I.N.I. measure anxiety and depression (major) Neuroendocrinological measurement of cortisol concentration level in hair
Daneshkhah et al. 2017	Observational cross-sectional study	***n* = 200** *M*: 140 (70%) nn-*M*: 60 (30%)	WHOQOL-BREF (quality of life questionnaire)GHQ-28	Quality of psychological health Quality of social relations Quality of the environment Severity of somatisation Sleep disorders Social dysfunction, depression
Ahmed et al. 2017	Observational cross-sectional study	***n* = 204** *M*: 135 (66.2%) nn-*M*: 69 (33.8%)	Symptom Checklist 90 Revised (SCL-90-R)Global Severity IndexPositive Symptom Total Positive Symptom Distress Index	Somatisation, obsessive-compulsive disorder Interpersonal susceptibility Depression, anxiety, hostility, phobia, paranoia, psychotisme Appetite disorder, sleep disorder
Knipscheer et al. 2015	Observational cross-sectional study	***n* = 66** *M*:66 nn-*M*: -	Harvard Trauma Questionnaire (HTQ-30)Hopkins Symptom Checklist (HSCL-25)Cope-Easy (measuring coping styles)Lowlands Acculturation Scale (level of cultural adjustment)	PTSD Anxiety and depression Coping style Level of cultural adjustment
Khodabakhshi et al. 2012	Observational case control study	***n* = 200** *M*: 100 nn-*M*: 100	ENRICH marital satisfaction questionnaireGHQ-28 questionnaire	Severity of somatisation Sleep disturbance Social dysfunction Depression
Chibber 2010	Observational cohort study	***n* = 4800** *M*: 1842 (38.4%) nn-*M*: 2958 (61.6%)	Behrendt & Mortiz diagnostic criteria for PTSD Flashbacks of mutilation Mini International Neuropsychiatric interview Rey Memory test	Affective disorder divided into two categories: PTSD Other: anxiety and affective disorder, flashbacks of mutilation
**Mixed method study (quantitative data)**				
Lever et al. 2018	Observational retrospective study	***n* = 13** *M*:13 nn-*M*: -	Hopkins Symptom Checklist (HSCL-25)Harvard Trauma Questionnaire revised-Part IV (HTQR-IV), for only 54% of people in the study	PTSD and traumatic life events Anxiety and depression Additional violence
Vloeberghs et al. 2012	Observational cross-sectional study	***n* = 66** *M*:66 nn-*M*: -	Harvard Trauma Questionnaire (HTQ-30)Hopkins Symptom Checklist (HSCL-25)Cope-Easy (measuring coping styles)Lowlands Acculturation Scale (level of cultural adjustment)	PTSD Anxiety/depression Coping style Level of cultural adaptation (integration, assimilation, separation and marginalisation)

*Source:* Please see the full reference list of the article for more information

*M*, mutilated women; nn-*M*, non-mutilated women; PTSD, post-traumatic stress disorder; WHOQOL-BREF, World Health Organization quality of life-BREF.

**TABLE 2:** Summary of qualitative and mixed method studies’ characteristics.

Authors and date	Study design	Research question	Data collect method	Data analysis method	outcomes
**Qualitative study**					
Omigbodun et al. 2019	Observational descriptive qualitative study	Identification of FGM main meanings and perceptions of FGM/C psychological consequences according to women interviewed experiences	Free listing according to Fiks et al. 2011: structured interviews in subgroups	Thematic analysis + response frequencies study with ATLAS.ti	Community perceptions of FGM by participants’ experience of health, psychological and life impacts; by age, residential setting and by mutilated/non-mutilated status
Ahmed et al. 2019	Observational descriptive qualitative study	Knowledge, beliefs and attitudes of a Kurdish women sample on FGM	Group interviews: questions asked according to a ‘thematic guide’	Thematic analysis	Women’s perceptions on the different aspects of FGM: state of knowledge on the procedures of the practice, revelations of the devastating psychological and painful consequences of FGM, sexual consequences, link with religion, socio-cultural and couple issues
Jacobson et al. 2018	Observational descriptive qualitative study	Everyday life experience and body sensations of Somali-Canadian women with disabilities	Semi-structured individual interviews	Thematic and interpretative analysis according to Sadala and Adorno 2002	Experience of FGM in everyday life and body sensations according to: (1) feeling normal or stigmatised (2) resignation to undergo FGM (3) comparison to non-mutilated and Canadian women (4) pain and pleasure
Parikh et al. 2018	Observational descriptive qualitative study	Psychological effects of FGM in the United Kingdom	Semi-structured individual interviews: semi-open questions + specific questions	Thematic analysis according to Braun and Clarke 2006	Psychological effects according to (1) experience of FGM (2) social and family relationships (3) culture (4) supportive environment
**Mixed method study (qualitative data)**					
Lever et al. 2018	Observational descriptive retrospective qualitative study	What are mutilated women asylum seeker experiences of gender-based violence in the United States?	Collection of data from affidavits (sworn statements) of women and/or doctors at the centre in response to standardised questions	Constant comparison analysis by Sandelowski 2006	Types of gender-based violence experienced by women asylum seekers, in addition to FGM
Vloeberghs et al. 2012	Observational descriptive participative qualitative study	Does FGM lead to psychological, social and/or relationship problems? What is the nature of these problems? What factors contribute to the development of these problems? What are the coping mechanisms of immigrant women in the face of these problems?	Semi-structured individual interviews; based on list of themes developed by researchers and community members	Thematic analysis + study of frequencies, means, standard deviation of responses with ATLAS ti	FGM experience (type, memories of event, pre-FGM discussion, education about FGM); psychological problems; Social and sexual relationships; General health and caregivers; coping mechanisms

*Source:* Please see the full reference list of the article for more information

FGM, female genital mutilation; FGM/C, female genital mutilation or circumcision.

### Quality appraisal

The 14 included studies (eight quantitative, four qualitative and two mixed method) met the two basic MMAT criteria (clear research question and data answering the questions).

Concerning the eight quantitative studies (Ahmed et al. 2017; Chibber, El-Saleh & El Harmi 2011; Daneshkhah et al. 2017; Im, Swan & Heaton 2020; Khodabakhshi Koolaee et al. 2012; Knipscheer et al. 2015; Köbach, Ruf-Leuschner & Elbert 2018; Piroozi et al. 2020), only two of them were deemed at low risk of bias in all five MMAT categories and four had at least two items deemed at high risk of bias. The data sample was limited in some studies. Two articles lacked details on sample inclusion and/or exclusion criteria (Köbach et al. 2018; Piroozi et al. 2020). Five studies did not justify the sample size and four papers did not report the type of FGM/C experienced. The eight quantitative observational studies mainly used a validated tool (six out of eight studies).

Regarding the four qualitative studies, only one study was deemed at high risk of bias (Parikh, Saruchera & Liao 2020). Appropriate analysis methods were used (all papers used thematic analysis) and most of the other MMAT categories were respected (suitability of the data collection method with the research method; adequate data; interpretations substantiated by data; coherence between data, sources, collection, analysis and interpretation).

Finally, concerning the two mixed method studies (Lever et al. 2019; Vloeberghs et al. 2012), one study had four items deemed at high risk of bias (Lever et al. 2019). For example, it was not reported why they used a mixed method model for this study; quantitative and qualitative component were not combined to form a complete picture.

### Findings

#### Quantitative studies

Post-traumatic stress disorder, depression, anxiety and somatisation were the most frequently assessed psychological disorders across the 10 quantitative and mixed studies.

Comparisons between groups showed that PTSD is statistically more severe in the FGM/C group than in the groups that were non-mutilated. Moreover, women with type III mutilation had more severe PTSD than women with type I and II. This observation is illustrated by Knipscheer et al. (2015) regression analysis, which showed that PTSD’s severity can be predicted by several factors including type III FGM/C (*p* < 0.01). Similarly, Köbach et al. (2018) regression analysis showed severe forms of type I and II FGM/C significantly influencing PTSD score (*p* < 0.01), exacerbated with the number of additional traumas. Im et al. (2020) showed similar results after adjusted PTSD analyses by checking age and poly-victimisation.

Concerning depression, three studies (Daneshkhah et al. 2017; Khodabakhshi Koolaee et al. 2012; Piroozi et al. 2020) used the GHQ-28 questionnaire to measure the impact of FGM/C on this psychological disorder. They showed mean (±standard deviation [s.d.]) scores for FGM/C (mostly type I) and non-mutilated groups of respectively 6.12 (±4.45) versus 4.60 (±4.46), *p* = 0.008, 4.87 (±4.70) versus 4.30 (±4.27), *p* = 0.414 and 4.68 (±4.58) versus 3.75 (±3.92), *p* = 0.125 (a score of six or more indicating the presence of severe depression as shown by Knipscheer et al. [2015] and Vloeberghs et al. [2012]).

The combined HSCL-25 depression and anxiety scores were used to compare psychological impact of FGM/C types (Type I: 34.50 [±7.59], Type II: 43.75 [±17.81] and Type III: 47.19 [±17.30]), knowing that a total score greater than or equal to 43.75 indicate the presence of depression and anxiety (Knipscheer et al. 2015; Vloeberghs et al. 2012). Regression analyses conducted by Ahmed et al. (2017); Knipscheer et al. (2015) and Piroozi et al. (2020), confirmed that mutilation (especially type III) is a factor influencing mental health scores and particularly depression.

Although anxiety was assessed differently in the nine studies (Ahmed et al. 2017; Chibber et al. 2011; Daneshkhah et al. 2017; Im et al. 2020; Knipscheer et al. 2015; Köbach et al. 2018; Lever et al. 2019; Piroozi et al. 2020; Vloeberghs et al. 2012), the analysis results showed significantly greater anxiety in mutilated groups except for Daneshkhah et al. (2017); *p* = 0.742 and Piroozi et al. (2020); *p* = 0.809, where the anxiety level was already high in the non-mutilated group. Again, Köbach et al.’s (2018) regression analysis showed that type II and III increased anxiety disorder severity.

Somatisation was measured in five studies (Daneshkhah et al. 2017; Im et al. 2020; Köbach et al. 2018; Lever et al. 2019; Piroozi et al. 2020). It was significantly higher in mutilated than in non-mutilated women with *p*-values under 0.05 except for Daneshkhah et al. (2017) and Piroozi et al. (2020). Regression analyses conducted by Ahmed et al. (2017) showed that FGM/C was the only baseline factor that significantly influenced somatisation.

Further analysis revealed some predictive factors for PTSD, depression and anxiety disorders like vividness of FGM/C memory, use of illicit substances (Knipscheer et al. 2015), education about FGM/C and older age at the time of FGM/C.

#### Qualitative studies

These studies highlight the influence of socio-cultural and religious context on FGM/C psychological impact (stigmatisation and social isolation of non-mutilated women; and on the other hand, the feeling of belonging and access to marriage for mutilated women). These two factors also influence FGM/C practice perception (from the memory of a ‘horrible’ experience, especially for type III, to a feeling of relief, pride and hope for future social benefits). The western country immigrant status of some victims can also influence awareness about the consequences of FGM/C on sexual life, pain and daily activities. It can lead to feelings of anger, injustice and exclusion as well as awakening the sense that ‘something has been taken away’, of being ‘abnormal’ or inferior. Studies about immigrant women’s populations suggested participant coping mechanisms or emotional management in order to face FGM/C psychological consequences like using humour, listening to music, doing some physical activities, silence regarding the subject or forgiveness (Jacobson et al. 2018; Parikh et al. 2020; Vloeberghs et al. 2012).

## Synthesis of results

According to the JBI guidelines, the results of the quantitative, qualitative and mixed method studies were integrated into a ‘configurational’ analysis. While the quantitative studies found a significant relationship between FGM/C severity and four major psychological disorders (PTSD, depression, anxiety and somatisation), qualitative studies found contextual effects through victims’ accounts (immigrant’s status and socio-cultural background). There is consistent evidence, from several studies (Ahmed, Shabu & Shabila 2019; Jacobson et al. 2018; Parikh et al. 2020; Vloeberghs et al. 2012) of FGM/C impact on PTSD severity. Conversely, other elements were not considered in these studies such as mother/daughter relationship or discussing about FGM/C prior to its practice.

## Discussion

Even if the majority of quantitative studies establish a relationship between FGM/C severity and psychological disorders (PTSD, anxiety, depression, somatisation), some results are discordant. For example, although some studies used the same questionnaire, heterogeneity of the study populations should lead us to compare the results cautiously. For instance, Daneshkhah et al. (2017) did not detect the effect of FGM/C on depression or somatisation, most likely because of differences in demographic characteristics between the groups. Furthermore, in the Köbach et al. (2018) study, women with Type I FGM/C had depression scores similar to non-mutilated women. We hypothesised, with configurative analysis, that non-mutilated women in this study, who were of Somalian origin, may have experienced other traumas (stigmatisation, harassment, social isolation), which led to a high level of psychological distress in this group. The same phenomenon (high level of anxiety in the non-mutilated group) could explain the lack of difference between mutilated and non-mutilated women in the Piroozi et al. (2020) study.

The influence of cultural or ethnic origins was also illustrated by Knipscheer et al. (2015). They reported that being of Somalian origin (where Type III FGM/C is often practised) paradoxically attenuated PTSD severity, depression and anxiety disorders. Again, qualitative studies describing the social pressure and stigmatisation experienced by non-mutilated women (Ahmed et al. 2019; Jacobson et al. 2018; Omigbodun et al. 2020) in their community may shed light on this result. In this context, FGM/C may be experienced as a ‘rite of passage’ and confer positive psychological effects linked to social integration and benefits that arise from it.

Similarly, immigration generated a changing perception of FGM/C and higher levels of awareness among mutilated women through media, FGM/C abolition campaigns and the access to education which is available in host countries (Jacobson et al. 2018; Vloeberghs et al. 2012). This awareness triggered a feeling of ‘abnormal sense’ among these women, previously unheard of in their home countries where being mutilated was considered as ‘natural’ and ‘the order of things’ (Jacobson et al. 2018).

It is important to note that this ‘being abnormal’ feeling was sometimes awakened by an experience with health care (Jacobson et al. 2018): Vloeberghs et al. (2012) reported negative feelings (shame, embarrassment, guilt) caused by health professionals inappropriate behaviours creating a reluctance in some women to seek gynaecological care for example. Moreover, having to undress and show their genital area during clinical examination can trigger the memory of mutilation leading to PTSD symptoms similar to sexually abused or tortured women (Parikh et al. 2020; Vloeberghs et al. 2012). Considering the ‘silence’, mentioned by Jacobson et al. (2018) as a coping or emotional management mechanism, the need for a multidisciplinary approach for these women, based on a bio-psycho-social model as advocated by WHO (World Health Organization 2016) is obvious.

### Potential biases and limitations

As mentioned above, studying a heterogeneous population limits the interpretation of results and generalisability to larger populations. Despite filtering of eligibility criteria, selection bias and numerical imbalances between groups within the same study may also compromise the validity of some results.

#### Implications for practice

It is interesting to note that mutilated women suffer from chronic physical and psychological pain, which influence each other. Clinical management options could include a comprehensive clinical assessment including PTSD scales (e.g. Body Awareness Rating Scale), patient education on pain and PTSD mechanisms, relaxation techniques (meditation, music, relaxation, breathing, visualisation and distraction), pelvic floor rehabilitation and physical exercise.

Research on the mechanisms of pain in relation to the psychological repercussions in mutilated women could help to open new ways for the clinical management proposed to these patients. Treatment could be inspired by existing knowledge on the treatment of victims of sexual violence or PTSD.

## Conclusion

In conclusion, results of this mixed method systematic review reinforce the association of FGM/C (and its degree of severity) with psychological disorders such as PTSD, depression, anxiety and somatisation. It also illustrates contextual factors, including socio-cultural factors that may influence the intensity of these psychological disorders. This reinforces the need for multidisciplinary, culturally sensitive, specific and caring care for FGM/C victims.

Future research should develop adapted and standardised questionnaires to precisely study mutilated women’s psychological disorders. Studies should select comparable groups to the baseline to avoid confounding bias. Finally, research would benefit from mixed studies, as the combination of both quantitative and qualitative data would provide rich information close to clinical reality.
